# Are changes in radiological leg alignment and femoral parameters after total hip replacement responsible for joint loading during gait?

**DOI:** 10.1186/s12891-019-2832-5

**Published:** 2019-11-10

**Authors:** Stefan van Drongelen, Hanna Kaldowski, Timur Tarhan, Ayman Assi, Andrea Meurer, Felix Stief

**Affiliations:** 1 Orthopaedic University Hospital Friedrichsheim gGmbH, Dr. Rolf M. Schwiete Research Unit for Osteoarthritis, Marienburgstr. 2, 60528 Frankfurt/Main, Germany; 2 Orthopaedic University Hospital Friedrichsheim gGmbH, Frankfurt/Main, Germany; 3 Laboratory of Biomechanics and Medical Imaging, Faculty of Medicine, University of Saint-Joseph, Beirut, Lebanon

**Keywords:** Total hip replacement, Leg alignment, Unilateral hip osteoarthritis, Gait analysis, Joint loading

## Abstract

**Background:**

Gait kinematics after total hip replacement only partly explain the differences in the joint moments in the frontal plane between hip osteoarthritis patients after hip replacement and healthy controls. The goal of this study was to determine if total hip replacement surgery affects radiological leg alignment (Hip-Knee-Shaft-Angle, femoral offset, Neck-Shaft-Angle and varus/valgus alignment) and which of these parameters can explain the joint moments, additionally to the gait kinematics.

**Methods:**

22 unilateral hip osteoarthritis patients who were scheduled for total hip replacement were included in the study. Preoperatively and 1 year postoperatively all patients had biplanar radiographic examinations and 3D gait analysis.

**Results:**

The operated leg showed significantly (*P* < 0.05) more varus (1.1°) as well as a larger femoral offset (+ 8 mm) and a larger Hip-Knee-Shaft-Angle (+ 1.3°) after total hip replacement; however no significant differences in the joint moments in the frontal plane compared to healthy controls were found. The hip moment (first half of stance) and the knee moments (first and second half of stance) were mostly determined by the varus/valgus alignment (29% and respectively 36% and 35%). The combination with a kinematic parameter (knee range of motion, foot progression angle) increased the predictive value for the knee moments.

**Conclusion:**

In our patient group the joint moments after total hip replacement did not differ from healthy controls, whereas radiological leg alignment parameters changed significantly after the total hip replacement. A combination of these radiological leg parameters, especially the varus alignment, and the deviating kinematics explain the joint moments in the frontal plane during gait after total hip replacement surgery. For surgeons it is important not to create too much of a structural varus alignment by implanting the new hip joint as varus alignment can increase the knee adduction moment and the risk for osteoarthritis of the medial knee compartment.

**Trial registration:**

This study was retrospectively registered with DRKS (German Clinical Trials Register) under the number DRKS00015053. Registered 1st of August 2018.

## Background

It is known that the joint loads, expressed as net joint moments, do not return to normal up to 2 years after total hip replacement (THR) [1]. Patients still show a reduced external knee adduction moment in the second half of stance in both the operated leg and the non-operated leg compared to a healthy control group. Due to this lateralization of the knee joint load, patients likely have an increased risk for lateral knee osteoarthritis (OA) after THR [2, 3]. For the external hip adduction moments the literature is not unambiguous as some studies found increased hip loading [1], whereas other studies did not find an overload [4] for patients after THR.

Differences in gait kinematics between hip OA patients after THR and healthy controls can partly explain the differences in the joint moments in the frontal plane [1]. The decreased hip range of motion (RoM) in the sagittal plane was found to relate to the decreased knee adduction moment whereas the increased hip adduction angle was related to the increased hip adduction moment.

It is also known that leg alignment has an influence on the knee joint load: i.e. a varus alignment increases the medial knee joint load [5]. By performing a THR, the positioning of the prosthesis has an influence on the natural alignment of the leg. A slightly increased femoral offset (FO) is currently standard practice after THR, as a larger FO provides more stability, reduces wear [6, 7] and gives a better functional outcome [8]. A reduced FO creates an asymmetrical gait pattern with a reduced knee RoM on the affected side [8]. A recent simulation study of Rüdiger et al. [9] showed that a reduced FO increases abductor muscle force to maintain normal gait, which in turn increases the joint reaction force. Renkawitz et al. [10] found that patients with a restored FO after THR walk with more hip adduction and walk faster.

Ollivier et al. [11] showed that besides the larger FO, the leg also appeared to be more varus after THR. As mentioned, this change in leg alignment might increase the risk for medial knee OA since a varus alignment increases the knee joint load [5]. However, how other radiological leg alignment parameters change after THR and how these changes work out on the kinematics and kinetics of gait are not well documented [11, 12].

Up till now the preferred diagnostic system to verify the planning and correctness of fit of prosthesis is the conventional X-ray system: as such, 2D leg alignment parameters collected from conventional X-rays were correlated to gait data [8, 13, 14]. In contrast to conventional pelvic overview X-rays, which are taken with the patient in a supine position, biplanar EOS images are taken with the patient in an upright standing position of the entire lower limbs from pelvis to feet [15]. As such, from the EOS images 3D radiological parameters of the whole leg can be calculated to verify the fit of the prosthesis as well as to document changes in radiological leg parameters after THR. In hip OA patients, an increased sacral slope and an increased femoral mechanical angle have been found compared to healthy subjects [16]. These differences might be due to degenerative changes over time due to OA or inherent differences between individuals. To eliminate these influencing factors also data of healthy controls will be included in this study.

The goal of this study was to determine which changes in radiological leg alignment parameters (Hip-Knee-Shaft-Angle, femoral offset, Neck-Shaft-Angle and varus/valgus alignment) occur due to THR and which of these parameters can explain abnormal joint loads during gait. This leads to the following hypotheses: 1) A THR surgery affects radiological leg alignment parameters. 2) The knee and hip joint frontal moments during gait are related to radiological leg alignment parameters after THR.

## Methods

### Study design and protocol

This prospective study was carried out from May 2016 till February 2019. All subjects underwent a 3D gait analysis and biplanar radiographic examinations in a standing position (EOS®, EOS imaging SA, Paris, France). Patients had their gait analysis and radiographs performed preoperatively and 1 year postoperatively. The 3D model, reconstructed from the preoperative images by EOS imaging, was used by the operating surgeon for planning the prosthesis in the hipEOS® planning software. The surgery was performed by one of two experienced orthopaedic surgeons who both used a lateral surgical approach on all patients.

### Patients and healthy controls

 Twenty-seven symptomatic unilateral hip OA patients (10 male, 17 female), who were scheduled for THR were included in the study. Exclusion criteria were the inability to walk without walking aids, body mass index (BMI) > 30 kgm^− 2^, inflammatory arthritis, orthopaedic surgeries within the past 6 months and previous joint replacement in the lower extremities. Healthy control subjects were included if they had no history of orthopaedic surgeries or chronic and neuromuscular diseases.

All patients and healthy subjects gave informed consent prior to participation. Our institution’s medical ethics committee approved the study under the number 497/15. Patient gait data were compared to gait data of 15 healthy controls (9 male, 6 female) with a similar age distribution (Table 1). Patients’ preoperative and postoperative leg alignment data were compared to data of both legs of 53 healthy controls (26 male, 27 female) collected as part of a large study in Lebanon [17].

**Table 1:** Anthropometric data and walking speed of patients and healthy controls

	gait			leg alignment	
	patients postoperative (*N* = 22)	healthy controls (*N* = 15)		healthy controls (*N* = 53)	
Age (years)	62.3 (10.2)	61.5 (8.0)	*P* = 0.804	41.8 (8.2)	***P*** **< 0.001**
Height (m)	1.71 (0.10)	1.74 (0.09)	*P* = 0.353	1.69 (0.10)	*P* = 0.476
Weight (kg)	82.4 (16.7)	71.7 (14.7)	*P* = 0.053	74.3 (13.7)	***P*** **= 0.033**
BMI (kgm^−2^)	28.2 (4.9)	23.5 (2.9)	***P*** **= 0.002**	25.9 (3.5)	*P* = 0.060*
Speed (ms^−1^)	1.15 (0.14)	1.26 (0.09)	***P*** **= 0.009**		

Values are mean values with standard deviation in parenthesis. Comparison between patients and healthy controls with corresponding P-values (Independent-Samples T-Tests). BMI: body mass index; significant differences are bold printed. * Variances not equal (Levene’s Test)

### Radiographic measurements

**Fig. 1:**
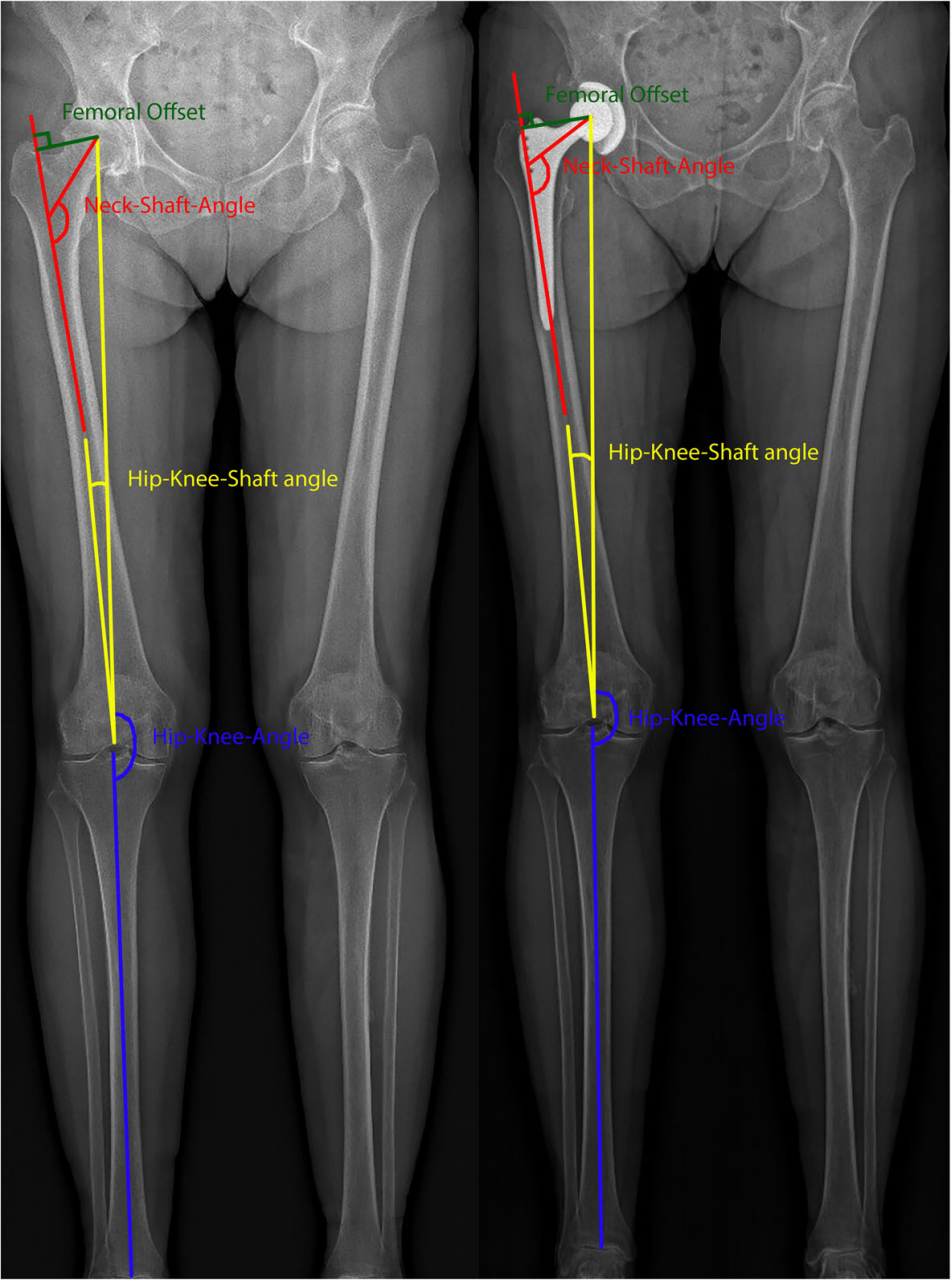
Schematic illustration of the leg alignment parameters projected on a preoperative (left) and postoperative (right) EOS image

Three-dimensional reconstruction of the lower limbs was performed for all subjects [18]. Four 3D radiological leg alignment parameters (including both the leg alignment and femoral parameters) were extracted from this reconstruction (Fig. 1):

Hip-Knee-Shaft-Angle (HKS): the frontal plane angle measured between the mechanical femoral axis and an axis running from the centre of the trochlea to the centre of the distal diaphysis.

Femoral offset (FO): the distance from the centre of rotation of the femoral head to a line bisecting the long axis of the femur.

Neck-Shaft-Angle (NSA): the angle measured between the femoral diaphysis axis and the axis going from the centre of the femoral head through the femoral neck.

Hip-Knee-Angle (HKA): is similar to the mechanical axis [19] and represents the varus/valgus configuration of the knee. The HKA is defined on the frontal plane by the hip, knee and ankle. A value greater than 180° equals a valgus alignment, a value smaller than 180° a varus alignment.

### Gait analysis

Patients and healthy controls walked barefoot at a self-selected speed in the gait laboratory. Kinematic data were collected using an 8-camera Vicon MX T10 motion capture system (VICON Motion Systems, Oxford, UK) operating at 200 Hz, while two AMTI force plates (Advanced Mechanical Technology, Inc., Watertown, MA, USA) were used to synchronously collect kinetic data at 1000 Hz. The marker protocol used for this study was a modified version of the Plug-in-gait model [20]. After the measurement, 3D marker trajectories were reconstructed, missing frames were filled and data was smoothed with a Woltring filter with MSE 10 using the Vicon Nexus software (version 2.5, VICON Motion Systems, Oxford, UK). In five trials with a clear foot-forceplate-contact, events were set to identify gait cycles. Kinematic and kinetic variables were obtained using inverse dynamics equations in which the center of the hip joint was calculated with a standardized geometrical prediction method [21]. For the healthy controls, data of the left leg was processed, since no significant differences were found in the discrete variables of interest between the left and right side. Data were exported to Matlab (version R2016b, The Mathworks Inc., Ismaning, Germany) and patterns, which were normalized over the gait cycle, were calculated. To represent the knee and hip joint load, the maximum external knee and hip adduction moment during the first (KAM_1 and HAM_1) and second (KAM_2 and HAM_2) phase of stance were determined for each trial and averaged over the trials. All joint moments were normalized to body mass and expressed in Nmkg^− 1^. Kinematic parameters, which are known to influence the joint load [1, 22], were extracted as well: mean foot progression angle in the transverse plane (FPA: the angle of the long axis of the foot segment in the global coordinate system relative to the walking direction), maximum knee flexion and extension during stance, knee RoM in the sagittal plane (the difference between maximum flexion in the first half and the maximum extension during the second half of the stance), maximum lateral trunk displacement during stance (LTD: lateral displacement of the trunk with respect to the limb that has ground contact), maximum hip adduction angle during stance in the frontal plane, as well as the hip flexion, extension and RoM during stance in the sagittal plane. Walking speed was calculated as the mean value over the included trials.

### Statistical analysis

All data were analysed using IBM SPSS Statistics (version 25, IBM Deutschland GmbH, Ehningen, Germany). Shapiro-Wilk tests revealed normally distributed data. The radiological leg alignment parameters were investigated for differences between patients and controls via independent-samples Students’ t-tests and for differences between the affected/operated and non-affected/non-operated side in the patient group using paired-samples Students’ t-tests. For the non-operated leg, Cronbach’s α and mean differences between the preoperative and postoperative parameters were calculated to give information on the reliability of the measurement.

Walking speed was considered as a covariate to eliminate the effect of speed on the dependent kinematic and kinetic variables (ANCOVA: univariate analyses of variance with walking speed as a covariate and group as a fixed factor (operated leg or non-operated leg vs. controls)). Differences between the operated and non-operated side were investigated via paired-samples Students’ t-tests. Pearson’s correlation coefficients were calculated between the radiological leg alignment parameters and joint kinematics/joint kinetics, as well as among the radiological parameters themselves. For the patients, multiple regression analyses were performed with those parameters correlating significantly with the joint loads after THR. For all analyses *P*-values ≤ 0.05 were considered significant.

## Results

### Patients and healthy controls

Of the 27 patients, data of three patients could not be included: for one patient the preoperative EOS image could not be reconstructed, one EOS dataset was not valid due to movement artefacts and one gait analysis could not be used. Of the remaining 24 patients, two patients dropped out as they missed out on the post-operative gait analysis.

22 patients (9 male, 13 female) were included and apart from a significantly higher BMI (*P* = 0.002), the patients showed no significant differences to the data of 15 gait controls (Table 1). Patients walked significantly faster 1 year after surgery, however still significantly slower compared to the healthy controls (1.15 vs. 1.26 ms^− 1^, *P* = 0.009). For the comparison of the leg alignment parameters, the group of 53 controls was significantly younger (*P* < 0.001) and had a lower body weight compared to the patients (*P* = 0.033, Table 1).

### Radiological leg alignment parameters

For the non-operated leg, Cronbach’s α over 0.977 were found: mean differences 0.13 mm for the femoral offset and < 0.3° for the angles.

After THR the operated leg showed a larger FO (mean increase of 7.9 ± 6.7 mm), a decreased HKA, meaning the leg was put in more varus (1.1 ± 1.5°) and a larger HKS (1.3 ± 0.8°) compared to the situation before the THR (*P* < 0.002, Table 2). FO and HKS of the operated leg were postoperatively also significantly larger compared to the healthy controls and to the non-operated side (*P* < 0.001).

**Table 2:** Radiological leg alignment parameters for the affected/operated and non-affected/non-operated side

	preoperative		postoperative		healthy controls
	affected side	non-affected side	operated side	non-operated side	
HKS (°)	4.93 (1.27)	4.84 (1.23)	6.20 (1.22) ^***abc***^	4.90 (1.28)	4.53 (1.11)
FO (mm)	40.38 (8.17)	40.77 (7.54)	48.33 (9.64) ^***abc***^	41.08 (7.98)	39.44 (4.61)
NSA (°)	127.82 (5.86)	127.22 (5.79)	126.41 (7.84)	126.93 (5.47)	127.77 (4.20)
HKA (°)	179.09 (2.74)	178.70 (3.58)	177.96 (2.68) ^**b**^	178.83 (3.22)	179.09 (2.56)

*Values are mean values with standard deviation in parenthesis. Comparison between the affected/operated and non-affected/non-operated side (Paired-Samples T-Tests); comparison between the affected/operated/non-affected/non-operated side and healthy controls (Independent-Samples T-Tests). HKS = Hip-Knee-Shaft-Angle; FO = Femoral Offset; NSA = Neck-Shaft-Angle; HKA = Hip-Knee-Angle*

^***a***^ *significant difference between the affected and non-affected / operated and non-operated side*

^***b***^ *significant difference between the preoperative and postoperative value*

^***c***^ *significant difference to the healthy controls*

### Joint moments

The preoperative joint moments were reported in supplementary material (Table 5 in Appendix 1). For the postoperative joint moments no significant differences could be found between patients (operated and non-operated leg) and healthy controls (Table 3). Walking speed had a significant effect on KAM_1 and KAM_2 of both the operated and non-operated leg. Elimination of this effect did not cause a group effect (Table 3). No effect of speed could be detected for the hip adduction moments. A significant difference was found between HAM_1 of the operated and non-operated leg (*P* = 0.025).

**Table 3:** Postoperative kinetics and kinematics during stance phase for the operated side, non-operated side and healthy controls

	operated side	non-operated side	healthy controls	operated vs. non-operated	operated vs. healthy controls	non-operated vs. healthy controls
Kinetics						
KAM_1 (Nmkg^− 1^)	0.42 (0.16)	0.40 (0.18)	0.45 (0.16)	*P* = 0.590	*P* = 0.436*	*P* = 0.945*
KAM_2 (Nmkg^− 1^)	0.36 (0.13)	0.37 (0.15)	0.43 (0.14)	*P* = 0.737	*P* = 0.670*	*P* = 0.704*
HAM_1 (Nmkg^− 1^)	0.80 (0.15)	0.88 (0.18)	0.90 (0.21)	***P*** **= 0.025**	*P* = 0.379	*P* = 0.904
HAM_2 (Nmkg^− 1^)	0.76 (0.17)	0.73 (0.16)	0.76 (0.23)	*P* = 0.451	*P* = 0.601	*P* = 0.678
Kinematics						
FPA (°)	−10.3 (5.1)	−8.6 (4.5)	−5.9 (3.2)	*P* = 0.198	*P* = 0.056	*P* = 0.119
KneeFlex (°)	17.9 (5.3)	19.9 (5.8)	21.1 (3.9)	***P*** **= 0.029**	*P* = 0.085	*P* = 0.943
KneeExt (°)	6.3 (6.1)	5.3 (6.3)	3.2 (3.4)	*P* = 0.380	*P* = 0.651*	*P* = 0.662
KneeRoM (°)	11.6 (5.0)	14.6 (6.3)	17.8 (4.3)	***P*** **= 0.004**	***P*** = 0.013*	*P* = 0.607*
LTD (°)	−3.0 (2.0)	−1.3 (2.2)	−1.9 (1.9)	*P* = 0.055	*P* = 0.243	*P* = 0.614
HipAdd_1 (°)	6.4 (3.9)	6.0 (5.8)	6.8 (5.0)	*P* = 0.688	*P* = 0.944	*P* = 0.428
HipAdd_2 (°)	6.3 (2.7)	4.1 (4.3)	4.7 (4.8)	***P*** **= 0.034**	*P* = 0.300	*P* = 0.437
HipFlex (°)	31.9 (6.7)	33.6 (7.0)	33.9 (5.7)	***P*** **= 0.042**	*P* = 0.472	*P* = 0.748
HipExt (°)	−7.7 (9.7)	−9.1 (9.8)	−11.0 (7.1)	*P* = 0.344	*P* = 0.445	*P* = 0.713
HipRoM (°)	38.0 (5.4)	41.4 (7.5)	43.7 (3.7)	***P*** **= 0.045**	***P*** **= 0.014**	*P* = 0.848

Mean values with standard deviation in parenthesis. Comparison between operated and non-operated side (Paired-Samples T-Tests) as well as between operated/non-operated side and healthy controls (Univariate Analysis of Variance). KAM_1 = maximum external knee adduction moment in first phase of stance; KAM_2 = maximum external knee adduction moment in second phase of stance; HAM_1 = maximum external hip adduction moment in first phase of stance; HAM_2 = maximum external hip adduction moment in second phase of stance; FPA = mean foot progression angle in the transverse plane (external rotation (−)); KneeFlex = maximum knee flexion during stance (flexion (+) / extension (−)); KneeExt = maximum knee extension during stance (flexion (+) / extension (−)); KneeRoM = knee range of motion in the sagittal plane during stance; LTD = maximum lateral trunk displacement in the frontal plane during stance (towards the affected stance limb (−)); HipAdd_1 = maximum hip adduction during first phase of stance (adduction (+)); HipAdd_2 = maximum hip adduction during second phase of stance (adduction (+)); HipFlex = maximum hip flexion during stance (flexion (+) / extension (−)); HipExt = maximum hip extension during stance (flexion (+) / extension (−)); HipRoM = hip range of motion in the sagittal plane during stance; significant differences are bold printed;* significant effect of speed

### Joint kinematics

Like the joint kinetics, the preoperative joint kinematics were reported in supplementary material (Table 5 in Appendix 1). Patients walked with a smaller maximum knee flexion during stance compared to the non-operated leg (*P* = 0.029) which resulted in a reduced range of motion during stance compared to both the non-operated leg (*P* = 0.004) and the healthy controls (*P* = 0.013, Table 3). The operated hip showed a higher peak adduction angle compared to the non-operated hip during the second phase of stance (*P* = 0.034). Regarding the hip kinematics in the sagittal plane, patients walked with a lower maximum hip flexion compared to the non-operated leg (*P* = 0.042) which resulted in a reduced range of hip motion during stance compared to both the non-operated leg (*P* = 0.045) and the healthy controls (*P* = 0.014).

### Correlations

Here only the correlations for the operated leg data are presented (Table 4), the correlations between the joint moments, joint kinematics and the radiological leg alignment parameters for the non-operated leg (postoperatively) were reported in supplementary material (Table 6 in Appendix 2).

**Table 4:** Correlations between joint moments, joint kinematics and clinical leg parameters for the operated leg

	FO	HKA	HKS	NSA	FPA	KneeFlex	KneeExt	KneeRoM	LTD	HipAdd1	HipAdd2	HipFlex	HipExt	HipRoM
**KAM_1**	**0.439**	**− 0.599**	0.373	− 0.195	0.349	0.168	−0.231	**0.459**	**0.445**	−0.091	−0.096	0.142	0.077	0.170
	***P* = 0.041**	***P* = 0.003**	*P* = 0.088	*P* = 0.384	*P* = 0.112	*P* = 0.456	*P* = 0.301	***P* = 0.032**	***P*** **= 0.038**	*P* = 0.689	*P* = 0.671	*P* = 0.527	*P* = 0.734	*P* = 0.449
**KAM_2**	0.365	**−0.590**	0.181	0.025	**0.470**	0.059	−0.287	0.412	**0.496**	−0.132	−0.148	0.119	0.095	0.126
	*P* = 0.095	***P* = 0.004**	*P* = 0.420	*P* = 0.911	***P* = 0.027**	*P* = 0.794	*P* = 0.196	*P* = 0.057	***P* = 0.019**	*P* = 0.558	*P* = 0.512	*P* = 0.597	*P* = 0.675	*P* = 0.575
**HAM_1**	0.030	**−0.541**	0.135	0.062	−0.002	−0.131	− 0.212	0.120	0.240	0.409	0.117	0.258	0.086	0.199
	*P* = 0.893	***P* = 0.009**	*P* = 0.548	*P* = 0.783	*P* = 0.994	*P* = 0.561	*P* = 0.344	*P* = 0.594	*P* = 0.282	*P* = 0.059	*P* = 0.603	*P* = 0.247	*P* = 0.704	*P* = 0.375
**HAM_2**	0.089	−0.372	0.142	−0.174	0.150	−0.172	− 0.216	0.082	0.243	0.143	0.280	0.002	0.003	0.071
	*P* = 0.695	*P* = 0.088	*P* = 0.528	*P* = 0.440	*P* = 0.506	*P* = 0.443	*P* = 0.334	*P* = 0.717	*P* = 0.275	*P* = 0.525	*P* = 0.206	*P* = 0.992	*P* = 0.988	*P* = 0.754
**FO**	1	−0.398	0.331	**−0.500**	0.141	**0.440**	0.097	0.345	0.374	−0.290	−0.103	0.400	0.269	0.098
		*P* = 0.067	*P* = 0.132	***P* = 0.018**	*P* = 0.531	***P* = 0.041**	*P* = 0.666	*P* = 0.116	*P* = 0.086	*P* = 0.190	*P* = 0.649	*P* = 0.065	*P* = 0.226	*P* = 0.666
**HKA**	−0.398	1	−0.365	0.189	0.040	**−0.496**	−0.282	− 0.178	**−0.615**	0.278	0.317	**−0.587**	−0.418	− 0.099
	*P* = 0.067		*P* = 0.095	*P* = 0.400	*P* = 0.860	***P* = 0.019**	*P* = 0.203	*P* = 0.428	***P* = 0.002**	*P* = 0.211	*P* = 0.150	***P* = 0.004**	*P* = 0.056	*P* = 0.662
**HKS**	0.331	−0.365	1	**−0.440**	− 0.069	0.329	0.281	0.004	0.079	−0.209	−0.105	0.092	0.064	0.058
	*P* = 0.132	*P* = 0.095		***P* = 0.041**	*P* = 0.761	*P* = 0.134	*P* = 0.205	*P* = 0.984	*P* = 0.725	*P* = 0.351	*P* = 0.641	*P* = 0.685	*P* = 0.779	*P* = 0.799
**NSA**	**−0.500**	0.189	**−0.440**	1	0.329	**−0.467**	−0.330	− 0.090	−0.160	0.418	0.170	−0.276	−0.228	0.052
	***P* = 0.018**	*P* = 0.400	***P* = 0.041**		*P* = 0.135	***P* = 0.028**	*P* = 0.134	*P* = 0.690	*P* = 0.477	*P* = 0.053	*P* = 0.449	*P* = 0.214	*P* = 0.308	*P* = 0.820

*Pearson correlations and P values for the correlations between joint moments, joint kinematics and clinical leg parameters for the operated leg*

*Abbreviations: KAM_1 = maximum external knee adduction moment in first phase of stance; KAM_2 = maximum external knee adduction moment in second phase of stance; HAM_1 = maximum external hip adduction moment in first phase of stance; HAM_2 = maximum external hip adduction moment in second phase of stance; FO = Femoral Offset; HKA = Hip-Knee-Angle; HKS = Hip-Knee-Shaft-Angle; NSA = Neck-Shaft-Angle; FPA = mean foot progression angle in the transverse plane; KneeFlex = maximum knee flexion during stance; KneeExt = maximum knee extension during stance; KneeRoM = knee range of motion in the sagittal plane during stance; LTD = maximum lateral trunk displacement in the frontal plane during stance; HipAdd_1 = maximum hip adduction during first phase of stance; HipAdd_2 = maximum hip adduction during second phase of stance; HipFlex = maximum hip flexion during stance; HipExt = maximum hip extension during stance; HipRoM = hip range of motion in the sagittal plane during stance; significant differences are bold printed*

#### Radiological leg alignment parameters

A significant correlation was found between FO and the NSA-angle (r = − 0.500, *P* = 0.018) and between the NSA-angle and the HKS (r = − 0.440, *P* = 0.041). A larger FO leads to a smaller NSA-angle and patients with a smaller NSA-angle had a larger HKS and vice versa.

#### Joint moments with radiological leg alignment parameters

HKA showed a significant correlation with KAM_1, KAM_2 and HAM_1 (r = − 0.599, *P* = 0.003; r = − 0.590, *P* = 0.004; r = − 0.541, *P* = 0.009 respectively). This means that the joint moments increase with a smaller HKA-angle, a more varus leg, or vice versa. KAM_1 showed a correlation to FO (r = 0.439, *P* = 0.041) which means that the knee joint moment in the first half of the stance phase increases with a larger FO and vice versa.

#### Joint moments with joint kinematics

KAM_1 showed a significant correlation with the knee RoM during stance (r = 0.459, *P* = 0.032), which means that the knee load is higher when patients are walking with more knee RoM. Both KAM_1 and KAM_2 showed a significant correlation with LTD (r = 0.445, *P* = 0.038; r = 0.496, *P* = 0.019) which means that the knee moments are higher when the thorax shows less lateral bending or vice versa. KAM_2 also showed a correlation to the FPA (r = 0.470, *P* = 0.027): the knee load increased with less outward rotation of the foot in the second half of the stance phase and vice versa. For HAM_2 no significant correlations to the joint kinematics were found.

#### Radiological leg alignment parameters with joint kinematics

FO, HKA and NSA all showed a significant correlation to the maximum knee flexion (r = 0.440, *P* = 0.041; r = − 0.496, *P* = 0.019; r = − 0.467, *P* = 0.028): persons with a larger FO, a more varus alignment and a smaller NSA angle walked with more knee flexion. Additionally correlations were found between the HKA angle and the LTD (r = − 0.615, *P* = 0.002) and between the HKA angle and the maximum hip flexion (r = − 0.587, *P* = 0.004), which means that patients with more varus legs walked with less lateral bending of the thorax and with more hip flexion.

### Regression model to explain the joint moments

The multiple regression analysis showed that the HKA explained 36% of the postoperative KAM_1 (R^2^ = 0.36; F = 11.20; *P* = 0.003). Adding the knee RoM increased the model significantly (ΔR^2^ = 0.13, ΔF = 4.75, *P* = 0.042) and explained 49% of KAM_1. Lateral bending of the thorax or the FO did not increase the model nor did it increase the appended model with the HKA and knee RoM any further.

For KAM_2, lateral bending of the thorax did not increase the model created by the HKA (R^2^ = 0.35; F = 10.69; *P* = 0.004). FPA did increase the model significantly (ΔR^2^ = 0.24, ΔF = 11.38, *P* = 0.003) and explained 59% of KAM_2.

For HAM_1, the HKA explained 29% of postoperative HAM_1 (R^2^ = 0.29; F = 8.288; *P* = 0.009). For HAM_2 no regression model was performed since no significant correlation was found between HAM_2 and any radiological or kinematical parameter.

## Discussion

Some changes in the radiological leg alignment parameters after THR are common and desired. However, how THR influences the total leg alignment and how these changes affect gait is largely unknown. Therefore, the goal of this study was to detect changes in radiological leg alignment parameters measured with the EOS system which could explain the joint load after THR. In this study no significant differences in the joint moments in the frontal plane compared to healthy controls were found, however after THR we found an increased varus alignment, an increased femoral offset and an increased Hip-Knee-Shaft-Angle. Further the hip moment (first half of stance) and the knee moments (first and second half of stance) showed strong correlations to the varus/valgus alignment.

The changes in leg alignment found in the present study are in accordance with Ollivier et al. [11]: a greater femoral offset and slightly more varus. It must be kept in mind that leg alignment was measured with standard radiography, whereas in this study the 3D EOS measurements were used. The restored or slightly increased offset is essential for a good functional result, improving the hip abductor moment lever-arm and implant stability [23, 24]. In the present study a trend (*P* = 0.067) was found between FO and the HKA-angle (Table 4), which might indicate that a large FO puts the leg in a more varus alignment, which can increase the risk for medial knee OA [25]. Our results show that preoperatively the patients’ legs did not differ from healthy controls, contrary to what was found previously by Bendaya et al. [16]. As the non-operated leg is assumed not to be a healthy leg and the leg alignment of this leg might have suffered under the increased loading due to a deviating gait pattern over a prolonged time, the non-operated leg is not a valid reference. It appears that in our study sample preoperatively no degenerative changes or inherent differences between patients and heathy controls were present and the differences postoperatively after THR are solely due to the THR.

Regarding the absolute joint moments, no significant differences could be found between the postoperative patients and the healthy controls. The smaller, but not significant lower knee and hip joint moments during the first half of stance in the patient group might be attributed to the higher walking speed of the healthy controls [26], however the univariate analyses of variance showed no group difference when the effect of walking speed was removed. Overall the hip joint moments are similar to the values reported in the review of Ewen [27], who showed that the peak hip abduction moment after THR are mostly lower but not significantly reduced compared to healthy controls.

The knee and hip RoMs in the sagittal plane remain reduced after THR compared to healthy controls, this stiff gait pattern might be the persistence pattern of the learned gait pattern of before the THR [1]. The knee RoM showed a significant effect of walking speed, whereas the hip RoM was not dependent on speed. A reduced hip RoM after THR compared to healthy controls, independent of walking speed, was also presented in the review paper of Kolk et al. [4].

### Effect of the radiological leg alignment parameters and kinematics on the frontal plane joint moments

The main goal of this study was to explore how the changed leg alignment affected the joint load after THR. Both the 1st and 2nd KAM were found to correlate to the HKA-angle in both the operated and non-operated leg (Table 4, Table 6 in Appendix 2). Stronger correlations were found for the operated leg, therefore the changed leg alignment might account partly for the increased knee adduction moment after THR compared to the preoperative condition. The effect of a varus leg on the knee adduction moment has been shown in previous literature [5, 28]. The kinematic parameters which correlate to KAM_1, an increased knee RoM and less lateral bending of the thorax, point in the direction of a healthier walking pattern and also tend to increase the knee joint load in the direction of the values of the healthy controls. These results are in accordance to previous studies [22, 29].

For KAM_2 the same conclusion could be drawn as for KAM_1, just that here the FPA, instead of knee RoM, showed a correlation. FPA is still more externally rotated postoperatively in THR patients, likely the result of the persistent learned compensation strategy to reduce the load [22]. An increase in FPA means that the foot orientation returns to normal which is accompanied with a higher KAM_2 as in the healthy control population. Overall, the larger part of the variation in KAM_1 and KAM_2 was explained by the varus alignment in combination with kinematic parameters returning to normal after THR.

Also HAM_1 was found to correlate to the HKA-angle (the varus alignment of the leg): legs more in varus showed higher external HAM_1 values. In contrast to the operated leg, the hip adduction moments of the non-operated leg can be explained for the greater part by the adduction angle. Also the variation of the adduction moment of the operated leg might be explained better by adding the hip adduction, which showed normal values but only a borderline significant correlation, to the regression equation. The relationship between the hip adduction and higher hip loads, expressed as hip contact forces, was shown in the study of Wesseling [30] who systematically investigated the effect of changed kinematics on hip contact forces.

Looking at the non-operated leg (Tables 2, 3), no significant differences were found regarding the joint moments, the kinematics and the radiological leg parameters compared to the healthy controls. The different correlations between the operated and non-operated leg could mean that the natural alignment is not preserved in the operated leg, as can also be seen in the correlations among the radiological leg parameters themselves. Stronger correlations were found between FO and NSA and between FO and the HKA angle for the non-operated leg.

As stated by Kolk et al. [4] differences between the operated and non-operated limbs are very interesting as it allows identifying compensatory inter-limb strategies. For the hip our results are similar to the results of Farkas et al. [31] who found lower hip RoM and lower hip adduction moments for the operated leg compared to the non-operated leg. Foucher and Wimmer showed that the differences between legs is important regarding the risk for OA, as for the knee higher adduction moments in the non-operated leg have been linked to the development [32] and aggravation of knee osteoarthritis [33]. In the present data however, a higher knee load in the non-operated leg compared to the operated leg could only be found preoperatively.

### Limitations

Of course other leg parameters which have not been analysed in this study might have an influence on the joint load during gait. Important factors when performing a THR are the torsion of the stem and leg length. These parameters were outside the scope of this research, but could have an effect on the changed joint moments and warrants further investigation.

Furthermore, the external joint moments only infer the joint loading during gait so that simulated contact forces or measured contact forces in vivo might show different results.

Components which could lead to a reduction in the peak abduction moment at the hip are muscle strength and surgical approach [27]. In this study a lateral surgical approach was used on all patients, but no detailed information on abductor muscle strength was collected postoperatively. Although the same surgical approach was used, different implants were used which could have had an effect on the overall leg alignment after surgery [34].

## Conclusions

In this study it was found that radiological leg alignment parameters changed significantly after THR. Whereas for our study group the joint moments after THR did not differ from healthy controls, the joint load showed a strong correlation to the varus alignment of the leg: patients with a more varus leg are likely to have higher knee and hip joint loads. A combination of this varus alignment, and the deviating kinematics (knee RoM, foot progression angle) explained the knee joint moments in the frontal plane during gait after THR surgery even better.

For surgeons it is not only necessary to minimize structural leg length inequality and to restore the femoral offset, it is also necessary not to create too much of a structural varus alignment by implanting the new hip joint as varus alignment can increase the risk for OA of the medial knee compartment.

## Data Availability

All data generated or analysed during this study are available upon reasonable request from the corresponding author.

## Appendix 1

**Table 5:** Preoperative kinetics and kinematics during stance phase for the affected side, non-affected side and healthy controls

	affected side	non-affected side	healthy controls	affected vs. non-affected	affected vs. healthy controls	non-affected vs. healthy controls
Kinetics						
KAM_1 (Nmkg^− 1^)	0.39 (0.17)	0.44 (0.13)	0.45 (0.16)	*P* = 0.119	*P* = 0.946	*P* = 0.410
KAM_2 (Nmkg^− 1^)	0.31 (0.13)	0.39 (0.12)	0.43 (0.14)	***P*** **= 0.002**	*P* = 0.062	*P* = 0.840
HAM_1 (Nmkg^− 1^)	0.91 (0.21)	0.98 (0.24)	0.90 (0.21)	*P* = 0.101	*P* = 0.318	*P* = 0.058
HAM_2 (Nmkg^− 1^)	0.84 (0.20)	0.91 (0.24)	0.76 (0.23)	*P* = 0.069	*P* = 0.130	***P*** **= 0.032**
Kinematics						
FPA (°)	−9.1 (5.5)	−8.8 (4.9)	−5.9 (3.2)	*P* = 0.794	*P* = 0.136	*P* = 0.368
KneeFlex (°)	16.7 (5.8)	19.5 (6.3)	21.1 (3.9)	*P* = 0.051	*P* = 0.134	*P* = 0.336*
KneeExt (°)	10.8 (7.2)	5.9 (6.4)	3.2 (3.4)	***P*** **< 0.001**	***P*** **= 0.025**	*P* = 0.671
KneeRoM (°)	5.9 (4.5)	13.6 (7.4)	17.8 (4.3)	***P*** **< 0.001**	***P*** **< 0.001***	*P* = 0.620*
LTD (°)	− 3.7 (2.4)	− 0.9 (2.4)	−1.9 (1.9)	***P*** **= 0.007**	*P* = 0.181	*P* = 0.531
HipAdd_1 (°)	7.2 (4.4)	6.0 (5.2)	6.8 (5.0)	*P* = 0.357	*P* = 0.556	*P* = 0.978
HipAdd_2 (°)	6.1 (4.1)	5.3 (4.0)	4.7 (4.8)	*P* = 0.475	*P* = 0.361	*P* = 0.691
HipFlex (°)	32.7 (10.6)	37.9 (10.9)	33.9 (5.7)	***P*** **< 0.001**	*P* = 0.885	*P* = 0.211
HipExt (°)	5.7 (14.8)	−5.7 (13.3)	−11.0 (7.1)	***P*** **< 0.001**	***P*** **= 0.013**	*P* = 0.784
HipRoM (°)	25.6 (7.7)	43.1 (9.4)	43.7 (3.7)	***P*** **< 0.001**	***P*** **< 0.001***	*P* = 0.104*

*Mean values with standard deviation in parenthesis. Comparison between affected and non-affected side (Paired-Samples T-Tests) as well as between affected/non-affected side and healthy controls (Univariate Analysis of Variance). KAM_1 = maximum external knee adduction moment in first phase of stance; KAM_2 = maximum external knee adduction moment in second phase of stance; HAM_1 = maximum external hip adduction moment in first phase of stance; HAM_2 = maximum external hip adduction moment in second phase of stance; FPA = mean foot progression angle in the transverse plane (external rotation (−)); KneeFlex = maximum knee flexion during stance (flexion (+) / extension (−)); KneeExt = maximum knee extension during stance (flexion (+) / extension (−)); KneeRoM = knee range of motion in the sagittal plane during stance; LTD = maximum lateral trunk displacement in the frontal plane during stance (towards the affected stance limb (−)); HipAdd_1 = maximum hip adduction during first phase of stance (adduction (+)); HipAdd_2 = maximum hip adduction during second phase of stance (adduction (+)); HipFlex = maximum hip flexion during stance (flexion (+) / extension (−)); HipExt = maximum hip extension during stance (flexion (+) / extension (−)); HipRoM = hip range of motion in the sagittal plane during stance; significant differences are bold printed; * significant effect of speed*

## Appendix 2

**Table 6:** Correlations between the joint moments, joint kinematics and the clinical leg parameters for the non-operated leg

	FO	HKA	HKS	NSA	FPA	KneeFlex	KneeExt	KneeRoM	LTD	HipAdd1	HipAdd2	HipFlex	HipExt	HipRoM
**KAM_1**	0.326	**− 0.431**	0.415	− 0.400	0.040	− 0.038	− 0.149	0.113	0.197	− 0.384	− 0.342	**0.449**	0.169	0.191
	*P* = 0.139	***P***** = 0.045**	*P* = 0.055	*P* = 0.065	*P* = 0.861	*P* = 0.868	*P* = 0.507	*P* = 0.616	*P* = 0.378	*P* = 0.077	*P* = 0.120	***P*** **= 0.036**	*P* = 0.451	*P* = 0.395
**KAM_2**	**0.488**	**− 0.438**	**0.471**	**− 0.535**	0.272	0.087	− 0.271	0.348	− 0.095	**−0.436**	− 0.400	0.326	− 0.033	0.319
	***P*** **= 0.021**	***P***** = 0.042**	*P*** = 0.027**	***P*** **= 0.010**	*P* = 0.220	*P* = 0.699	*P* = 0.223	*P* = 0.113	*P* = 0.673	***P*** **= 0.043**	*P* = 0.065	*P* = 0.138	*P* = 0.885	*P* = 0.148
**HAM_1**	−0.180	0.004	0.015	0.165	−0.063	− 0.165	0.253	− 0.401	0.317	**0.724**	**0.705**	0.086	0.262	−0.328
	*P* = 0.422	*P* = 0.987	*P* = 0.947	*P* = 0.463	*P* = 0.779	*P* = 0.462	*P* = 0.257	*P* = 0.064	*P* = 0.151	***P***** < 0.001**	***P***** < 0.001**	*P* = 0.703	*P* = 0.238	*P* = 0.136
**HAM_2**	0.010	0.008	−0.111	−0.003	0.118	0.089	0.392	−0.305	−0.089	0.333	0.401	0.079	0.222	−0.208
	*P* = 0.966	*P* = 0.973	*P* = 0.622	*P* = 0.989	*P* = 0.602	*P* = 0.693	*P* = 0.071	*P* = 0.167	*P* = 0.693	*P* = 0.130	*P* = 0.065	*P* = 0.726	*P* = 0.321	*P* = 0.352
**FO**	1	**−0.671**	0.388	**−0.904**	−0.207	0.385	0.161	0.194	−0.340	**−0.575**	**− 0.532**	**0.521**	0.254	0.191
		***P*** **= 0.001**	*P* = 0.074	***P***** < 0.001**	*P* = 0.356	*P* = 0.077	*P* = 0.474	*P* = 0.388	*P* = 0.122	***P*** **= 0.005**	***P*** **= 0.011**	***P***** = 0.013**	*P* = 0.254	*P* = 0.395
**HKA**	**−0.671**	1	−0.374	**0.716**	0.361	−0.320	−0.202	− 0.094	0.167	**0.448**	**0.424**	**−0.730**	−0.406	− 0.168
	***P***** = 0.001**		*P* = 0.086	***P***** < 0.001**	*P* = 0.099	*P* = 0.146	*P* = 0.368	*P* = 0.677	*P* = 0.458	***P***** = 0.036**	***P*** **= 0.049**	***P***** < 0.001**	*P* = 0.061	*P* = 0.454
**HKS**	0.388	−0.374	1	**−0.461**	− 0.038	0.080	0.050	0.024	0.245	−0.264	−0.270	0.269	0.243	−0.115
	*P* = 0.074	*P* = 0.086		***P*** **= 0.031**	*P* = 0.865	*P* = 0.722	*P* = 0.824	*P* = 0.916	*P* = 0.271	*P* = 0.236	*P* = 0.224	*P* = 0.226	*P* = 0.277	*P* = 0.609
**NSA**	**−0.904**	**0.716**	**−0.461**	1	−0.052	**−0.448**	− 0.121	−0.291	0.174	**0.501**	**0.529**	**−0.580**	−0.219	− 0.290
	***P***** < 0.001**	***P***** < 0.001**	***P***** = 0.031**		*P* = 0.818	***P***** = 0.036**	*P* = 0.591	*P* = 0.189	*P* = 0.439	***P***** = 0.018**	***P***** = 0.011**	***P***** = 0.005**	*P* = 0.328	*P* = 0.190

*Pearson correlations and P values for the correlations between joint moments, joint kinematics and clinical leg parameters for the non-operated leg*

*Abbreviations: KAM_1 = maximum external knee adduction moment in first phase of stance; KAM_2 = maximum external knee adduction moment in second phase of stance; HAM_1 = maximum external hip adduction moment in first phase of stance; HAM_2 = maximum external hip adduction moment in second phase of stance; FO = Femoral Offset; HKA = Hip-Knee-Angle; HKS = Hip-Knee-Shaft-Angle; NSA = Neck-Shaft-Angle; FPA = mean foot progression angle in the transverse plane; KneeFlex = maximum knee flexion during stance; KneeExt = maximum knee extension during stance; KneeRoM = knee range of motion in the sagittal plane during stance; LTD = maximum lateral trunk displacement in the frontal plane during stance; HipAdd_1 = maximum hip adduction during first phase of stance; HipAdd_2 = maximum hip adduction during second phase of stance; HipFlex = maximum hip flexion during stance; HipExt = maximum hip extension during stance; HipRoM = hip range of motion in the sagittal plane during stance; significant differences are bold printed*

## Abbreviations

BMI: Body mass index
FO: Femoral offset
FPA: Foot progression angle in the transverse plane
HAM_1: Maximum hip adduction moment in first phase of stance
HAM_2: Maximum hip adduction moment in second phase of stance
HKA: Hip-Knee-Angle
HKS: Hip-Knee-Shaft-Angle
KAM_1: Maximum knee adduction moment in first phase of stance
KAM_2: Maximum knee adduction moment in second phase of stance
LTD: Lateral trunk displacement during stance
NSA: Neck-Shaft-Angle
OA: Osteoarthritis
RoM: Range of motion
THR: Total hip replacement
